# Dynamic linear modeling of monthly electricity demand in Japan: Time variation of electricity conservation effect

**DOI:** 10.1371/journal.pone.0196331

**Published:** 2018-04-30

**Authors:** Keita Honjo, Hiroto Shiraki, Shuichi Ashina

**Affiliations:** 1 Global Environment and Natural Symbiosis Division, Center for Environmental Science in Saitama (CESS), Kamitanadare 914, Kazo, Saitama, 347-0115 Japan; 2 School of Environmental Science, The University of Shiga Prefecture, Hassaka 2500, Hikone, Shiga, 522-8533 Japan; 3 Center for Social and Environmental Systems Research, National Institute for Environmental Studies (NIES), Onogawa 16-2, Tsukuba, Ibaraki, 305-8506 Japan; Chongqing University, CHINA

## Abstract

After the severe nuclear disaster in Fukushima, which was triggered by the Great East Japan earthquake in March 2011, nuclear power plants in Japan were temporarily shut down for mandatory inspections. To prevent large-scale blackouts, the Japanese government requested companies and households to reduce electricity consumption in summer and winter. It is reported that the domestic electricity demand had a structural decrease because of the electricity conservation effect (ECE). However, quantitative analysis of the ECE is not sufficient, and especially time variation of the ECE remains unclear. Understanding the ECE is important because Japan’s NDC (nationally determined contribution) assumes the reduction of CO_2_ emissions through aggressive energy conservation. In this study, we develop a time series model of monthly electricity demand in Japan and estimate time variation of the ECE. Moreover, we evaluate the impact of electricity conservation on CO_2_ emissions from power plants. The dynamic linear model is used to separate the ECE from the effects of other irrelevant factors (e.g. air temperature, economic production, and electricity price). Our result clearly shows that consumers’ electricity conservation behavior after the earthquake was not temporary but became established as a habit. Between March 2011 and March 2016, the ECE on industrial electricity demand ranged from 3.9% to 5.4%, and the ECE on residential electricity demand ranged from 1.6% to 7.6%. The ECE on the total electricity demand was estimated at 3.2%–6.0%. We found a seasonal pattern that the residential ECE in summer is higher than that in winter. The emissions increase from the shutdown of nuclear power plants was mitigated by electricity conservation. The emissions reduction effect was estimated at 0.82 MtCO_2_–2.26 MtCO_2_ (−4.5% on average compared to the zero-ECE case). The time-varying ECE is necessary for predicting Japan’s electricity demand and CO_2_ emissions after the earthquake.

## Introduction

**Fig 1:**
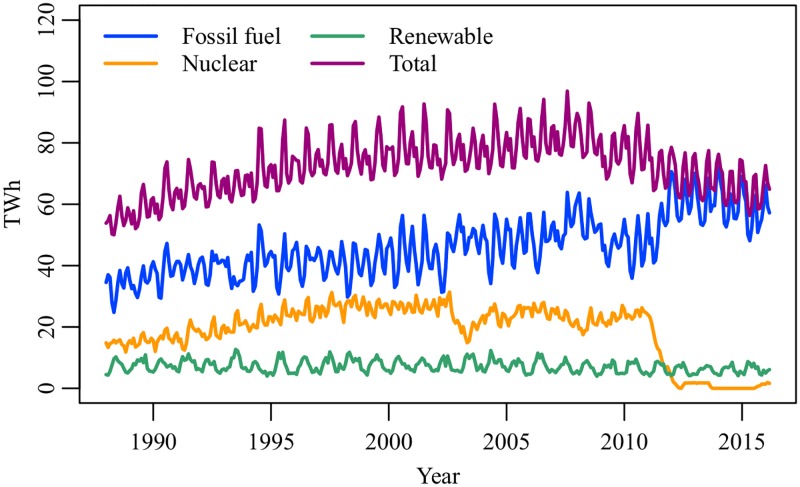
Japan’s electric power generation by energy source, January 1988–March 2016. Source: EDMC Databank [11].

After the severe nuclear disaster in Fukushima, which was triggered by the Great East Japan earthquake in March 2011, nuclear power plants in Japan were temporarily shut down for mandatory inspections. The Nuclear Regulation Authority requested electric power companies to ensure that all nuclear power plants satisfy new regulatory standards [1]. As of February 2018, only five reactors are in operation, nine reactors are preparing to resume operations, while 28 reactors remain closed [2]. To avoid a lack of power supply, electric power companies were forced to increase fossil fuel power generation (Fig 1). At the same time, the Japanese government asked companies and households to reduce electricity consumption in summer and winter. In particular, large-scale consumers (contracts of more than 500 kW) in the Kanto and Tohoku regions were obligated to reduce peak-time electricity consumption in the summer of 2011 by 15% compared to the summer of 2010 [3]. Early questionnaire surveys show that companies and households conserved electricity by turning off lights, introducing LED lights, and limiting the use of air conditioners [4–8]. Owing to the aggressive electricity conservation, Japan could prevent large-scale blackouts except for the rolling blackout implemented in the Kanto region right after the earthquake. In 2016 and 2017, the Japanese government did not request electricity conservation to consumers because the risk of electricity shortage was sufficiently low [9, 10].

Understanding the electricity conservation effect (ECE) is important from the viewpoint of climate policy. Japan’s NDC (nationally determined contribution), which was submitted to UNFCCC in July 2015, assumes the reduction of CO_2_ emissions through aggressive energy conservation [12]. According to METI [13], Japan needs to reduce electricity demand in 2030 by 17% compared to the BAU case. Since the earthquake, many researchers have investigated the ECE using econometric models. Cho et al. [14] estimate the annual ECEs for 47 prefectures of Japan using the spatial Durbin model. They obtain the result that the earthquake decreased the domestic electricity demand in 2011 by 1.3% and increased the domestic CO_2_ emissions in the same year by 0.3%. Hayashida et al. [15] estimate a regression model of quarterly electricity demand and compare electricity demand after the earthquake with the 2010 level. The ECE on industrial electricity demand in 2011 was 5.4%, and it increased to 7.1% in 2012. The ECE on the residential electricity demand increased from 5.5% to 9.9%. Nishio [7] investigates electricity conservation by households in the Kanto and Kansai regions using panel data collected by questionnaire surveys. In the summers of 2011–2014, the ECE in the Kanto region ranged from 8.8% to 11.2%, while the ECE in the Kansai region ranged from 6.0% to 11.7%. Kabe [16] estimates a regression model of annual electricity demand in the residential sector, and pointed out the possibility that electricity conservation after the earthquake was partly motivated by the rise in electricity price. Mase and Hayashida [17] estimate a regression model of quarterly electricity demand in the industrial sector. They apply the Wald test to the model and find that the production elasticity of electricity demand had a structural change in the summer of 2011.

Previous studies help us understand the impact of the earthquake on electricity demand, but they have three limitations. First, previous studies use annual or quarterly data, and the data size after the earthquake is quite small. Time variation of the ECE has not yet been sufficiently analyzed, and it remains unclear whether consumers’ electricity conservation behavior after the earthquake became established as a habit or not. Second, previous studies use static regression models (SRMs) with constant parameters and pay little attention to long-term changes in electricity consumption behavior of companies and households. Time series data of energy demand contain a trend driven by changes in technologies and consumers’ habits, which is called underlying energy demand trend (UEDT). It is known that the UEDT follows a stochastic process rather than a deterministic process [18–23]. Furthermore, the coefficients of explanatory variables (e.g. energy price, income, and air temperature) also stochastically change with time [20, 22, 24–26]. Due to the non-stationarity of electricity demand data, the SRM fails to separate the ECE from the effects of other irrelevant factors. Third, the estimation of the ECE by the SRM causes the overfitting problem. The SRM requires a dummy variable to estimate the ECE at each time step [7, 15]. The number of dummy variables increases as the data size after the earthquake increases. If the same approach is applied to monthly electricity demand, the model is overparameterized, and the estimation result becomes unreliable.

In this study, we develop a time series model of Japan’s monthly electricity demand and estimate time variation of the ECE. At the same time, we evaluate the reduction of CO_2_ emissions achieved by electricity conservation. To overcome the limitations of previous studies, we use the dynamic linear model (DLM) [27–30] instead of the SRM. The DLM, which is a natural expansion of the SRM, has time-varying parameters which follow stochastic processes (e.g. Gaussian random walk). The DLM has an advantage that it can detect stochastic trends hidden in time series data. The DLM with the time-varying intercept is helpful in estimating the UEDT, and it has been applied to energy demand data in various countries [18, 19, 21–23]. If time series data have cyclic patterns (e.g. monthly electricity demand), seasonal components are added to the model [20, 31–33]. The DLM is also used to investigate whether price and income elasticities of energy demand are time-varying or not [22, 24, 25]. Another advantage of the DLM is that it can easily describe structural changes in time series data. By introducing an intervention variable [30, 34] into the model, we can estimate the time-varying effect of an exogenous shock without inflating the number of parameters. These characteristics of the DLM are suitable for our research purpose.

In addition to the DLM, the artificial neural network (ANN) is also a powerful tool for predicting time variation of electricity demand. The ANN is the learning model which converts input vectors to output vectors using the network structure called hidden layer. The ANN requires less mathematical restrictions compared to statistical models (e.g. SRM and DLM) and can predict complex time series such as hourly electricity demand with high accuracy [35, 36]. Due to the network structure, however, it is difficult to extract interpretable information about the effects of explanatory variables from the estimated ANN. In contrast, we can easily interpret the estimation result of the DLM because the effects of explanatory variables are independent of each other. The ECE is directly given by the coefficient of the intervention variable which represents the impact of the earthquake on electricity demand. For this reason, the DLM is preferable to the ANN in the ECE estimation.

This paper is structured as follows. In Materials and Methods, the industrial and residential electricity demand models are defined. The model structure is determined based on Akaike information criterion (AIC) [30, 37]. In Results and Discussion, the estimation results of the electricity demand models are shown. The monthly ECEs are calculated, and the impacts of electricity conservation on electricity-related CO_2_ emissions are evaluated. Limitations of our approach are also discussed. Conclusion summarizes this paper.

## Materials and methods

### Electricity demand in Japan

**Fig 2:**
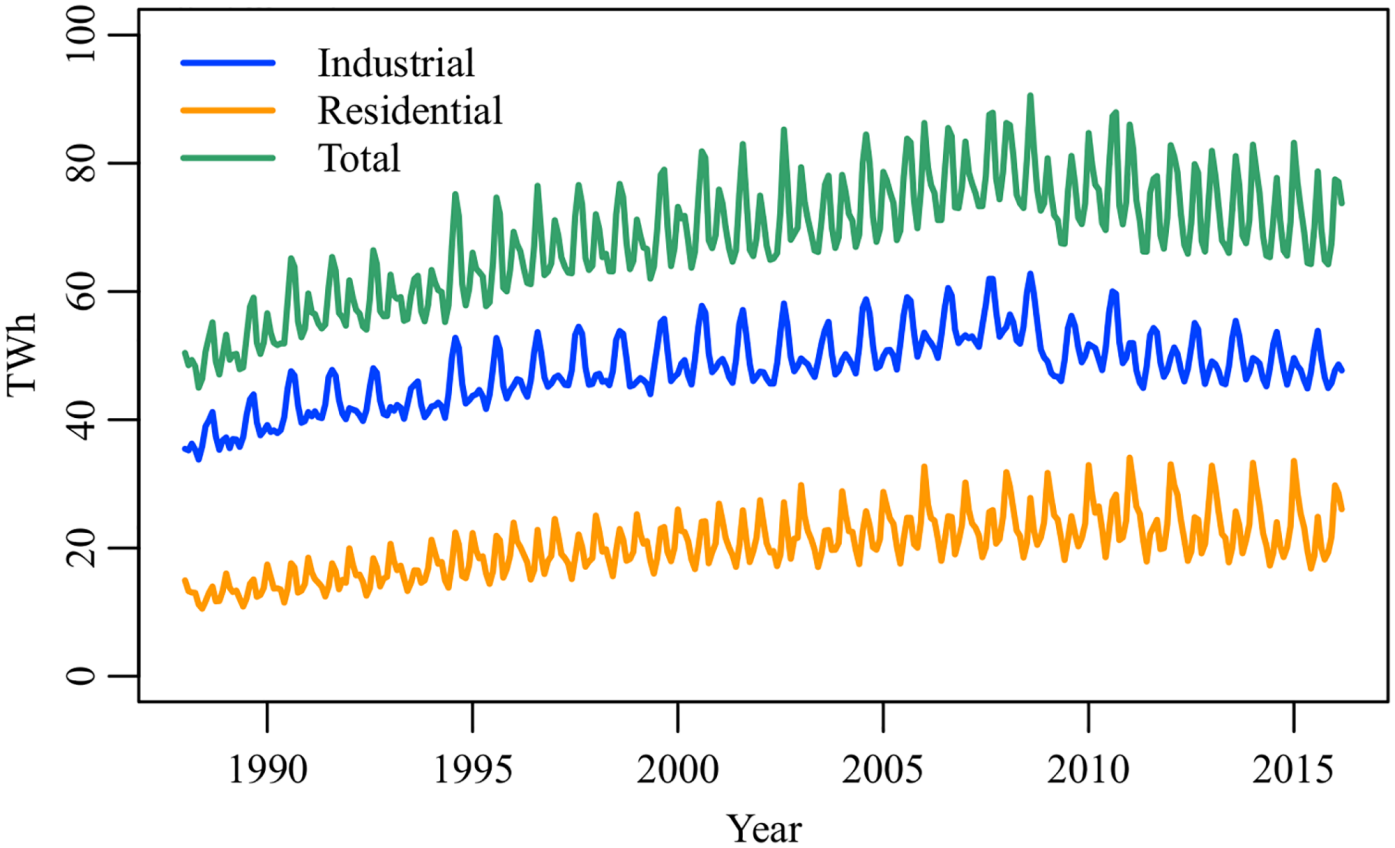
Industrial, residential, and total electricity demands in Japan, January 1988–March 2016. Source: EDMC Databank [11]. Self-consumption in companies with private power plants is not included.

In Japan, electricity demand is traditionally classified into two categories: Doryoku and Dento. The former indicates electricity used for large equipment of factories and office buildings (e.g. industrial motors, pumps, and elevators), while the latter indicates electricity used for small appliances (e.g. personal computers, packaged air conditioners, and room lights). For simplicity, we refer to Doryoku and Dento as the industrial and residential electricity demands, respectively. Fig 2 shows the industrial, residential, and total electricity demands between January 1988 and March 2016. The total electricity demand increased by 60% between 1988 and 2008, but no clear increase was observed in recent years. The industrial electricity demand accounts for 60%–78% of the total electricity demand. It has a seasonal peak in summer and is influenced by changes in economic production. A rapid fall caused by the 2008–2009 global financial crisis is seen. Meanwhile, the residential electricity demand has two seasonal peaks in summer and winter. The level of winter has continued to increase since 1988, and the seasonal difference is expanding. As the industrial and residential electricity demands show different cyclic patterns, we need to analyze them separately.

### Model equations

A DLM consists of an observation equation and state equations. The observation equation describes the relationships between electricity demand and explanatory variables. As explanatory variables, statistical models of electricity demand include economic activity indices (e.g. GDP, disposable income, and household consumption expenditure), energy prices, and weather conditions. We consulted previous studies about Japan’s electricity demand [15–17] and selected the explanatory variables such that long-term monthly data are available. Unlike the SRM, parameters can vary with time. The dynamics of parameters are described by state equations. To simplify the process of model estimation, we assume that parameters follow Gaussian random walks. In this case, parameters can be estimated by combining the maximum likelihood estimation and the Kalman filter. The R package *dlm* [29, 38] is used for model estimation.

#### Industrial electricity demand

The observation equation of industrial electricity demand ($E_{t}^{\text{ind}}$) is defined as

$$\begin{array}{c} \log E_{t}^{\text{ind}}=\sigma_{t}^{\text{ind}}+\theta_{t}^{1}+\theta_{t}^{2}C_{t}+\theta_{t}^{3}H_{t}+\theta_{t}^{4}\log Q_{t}+\theta_{t}^{5}\log P_{t}^{\text{ind}}+\theta_{t}^{6}I_{t}+v_{t}^{\text{ind}}, \end{array}$$ (1)

where

$$\begin{array}{c} v_{t}^{\text{ind}}∼N\left(0,V^{\text{ind}}\right). \end{array}$$ (2)

*C*_*t*_ and *H*_*t*_ are cooling and heating degree days (CDD and HDD), respectively. Let $T_{t}^{1},T_{t}^{2},\ldots ,T_{t}^{n(t)}$ be the data of daily average temperature (DAT) in month *t*. Then

$$\begin{array}{c} C_{t}=\sum_{i=1}^{n(t)}\max\left\{T_{t}^{i}-T_{\text{cdd}}^{*},0\right\}, \end{array}$$ (3)

$$\begin{array}{c} H_{t}=\sum_{i=1}^{n(t)}\max\left\{T_{\text{hdd}}^{*}-T_{t}^{i},0\right\}, \end{array}$$ (4)

where $T_{\text{cdd}}^{*}$ and $T_{\text{hdd}}^{*}$ are base temperatures. As the proxies of countrywide CDD and HDD, we use the population-weighted mean values of the degree-day indices in the central cities of 47 prefectures. The base temperatures differ from region to region because the demand for air conditioning depends on climatic conditions, types of buildings, and consumers’ lifestyle [39–41]. We consider the base temperatures as parameters and search the best combination through model selection (see Model selection). *Q*_*t*_ is an economic production index, and $P_{t}^{\text{ind}}$ is an electricity price index in the industrial sector. *I*_*t*_ is the intervention variable that switches from zero to one in March 2011. The seasonal component $\sigma_{t}^{\text{ind}}$ represents the basic electricity demand that is unique in each month. $\theta_{t}^{1}$ is the intercept, and $\theta_{t}^{2},\theta_{t}^{3},\ldots ,\theta_{t}^{6}$ are the coefficients of the explanatory variables. $v_{t}^{\text{ind}}$ is an observation error term with variance *V*^ind^.

The state equations of industrial electricity demand are defined as follows:

$$\begin{array}{c} \sigma_{t+1}^{\text{ind}}=-\sum_{i=1}^{11}\sigma_{t+1-i}^{\text{ind}}+s_{t}^{\text{ind}},\;s_{t}^{\text{ind}}∼N\left(0,S^{\text{ind}}\right), \end{array}$$ (5)

$$\begin{array}{c} \theta_{t+1}^{j}=\theta_{t}^{j}+w_{t}^{j},\;w_{t}^{j}∼N\left(0,W^{j}\right),\;j\in \left\{1,2,\ldots ,6\right\}. \end{array}$$ (6)

$s_{t}^{\text{ind}},w_{t}^{1},w_{t}^{2},\ldots ,w_{t}^{6}$ are state error terms with variances *S*^ind^, *W*^1^, *W*^2^, …, *W*^6^, respectively. From the signs of the state error variances (SEVs), we can know whether parameters are time-varying or not. If all the SEVs are zero, the DLM is equivalent to the SRM. Eq (5) describes the dynamics of the seasonal component. Monthly electricity demand has a cycle of 12 months. The static seasonal component is given by the combination of constants $\sigma_{\tau }^{\text{ind}},\sigma_{\tau +1}^{\text{ind}},\ldots ,\sigma_{\tau +11}^{\text{ind}}$ such that $\sum_{t=\tau }^{\tau +11}\sigma_{t}^{\text{ind}}=0$ for any *τ* ≥ 1. Hence $\sigma_{\tau +11}^{\text{ind}}=-\sum_{t=\tau }^{\tau +10}\sigma_{t}^{\text{ind}}$. This equality is rewritten as $\sigma_{t+1}^{\text{ind}}=-\sum_{i=1}^{11}\sigma_{t+1-i}^{\text{ind}}$ for any *t* ≥ 11. By adding the state error term to the right-hand side, we obtain Eq (5). See Durbin and Koopman [30] for details.

#### Residential electricity demand

The observation equation of residential electricity demand ($E_{t}^{\text{res}}$) is defined as

$$\begin{array}{c} \log(\frac{E_{t}^{\text{res}}}{N_{t}})=\sigma_{t}^{\text{res}}+\zeta_{t}^{1}+\zeta_{t}^{2}C_{t}+\zeta_{t}^{3}H_{t}+\zeta_{t}^{4}\log Y_{t}+\zeta_{t}^{5}\log P_{t}^{\text{res}}+\zeta_{t}^{6}I_{t}+u_{t}, \end{array}$$ (7)

where

$$\begin{array}{c} u_{t}∼N\left(0,U\right). \end{array}$$ (8)

*N*_*t*_ is population, *Y*_*t*_ is the real wage index, and $P_{t}^{\text{res}}$ is an electricity price index in the residential sector. To separate the ECE from the effect of population growth, we use residential electricity demand per capita as the response variable. $\sigma_{t}^{\text{res}}$ is the seasonal component, $\zeta_{t}^{1}$ is the intercept, and $\zeta_{t}^{2},\zeta_{t}^{3},\ldots ,\zeta_{t}^{6}$ are the coefficients of the explanatory variables. *u*_*t*_ is an observation error term with variance *U*. It is known that the day of the week (DOW) influences electricity demand in households through occupants’ activities [42–45]. To separate the ECE from the DOW effect, we add the following static regression term to the observation equation:

$$\begin{array}{c} u_{t}=\sum_{i=1}^{7}\eta^{i}D_{t}^{i}+v_{t}^{\text{res}},\;v_{t}^{\text{res}}∼N\left(0,V^{\text{res}}\right), \end{array}$$ (9)

where $D_{t}^{1},D_{t}^{2},\ldots ,D_{t}^{7}$ are the numbers of Mondays, Tuesdays, Wednesdays, Thursdays, Fridays, Saturdays, and Sundays in each month, respectively. *η*^1^, *η*^2^, …, *η*^7^ are constant parameters, and $v_{t}^{\text{res}}$ is an observation error term with variance *V*^res^. Not all of the DOW variables are necessary for predicting residential electricity demand. We select the combination of the DOW variables that minimizes AIC of the model (see Model selection).

Similar to the industrial electricity demand model, the state equations of residential electricity demand are defined as follows:

$$\begin{array}{c} \sigma_{t+1}^{\text{res}}=-\sum_{i=1}^{11}\sigma_{t+1-i}^{\text{res}}+s_{t}^{\text{res}},\;s_{t}^{\text{res}}∼N\left(0,S^{\text{res}}\right), \end{array}$$ (10)

$$\begin{array}{c} \zeta_{t+1}^{j}=\zeta_{t}^{j}+z_{t}^{j},\;z_{t}^{j}∼N\left(0,Z^{j}\right),\;j\in \left\{1,2,\ldots ,6\right\}, \end{array}$$ (11)

where $s_{t}^{\text{res}},z_{t}^{1},z_{t}^{2},\ldots ,z_{t}^{6}$ are state error terms, and *S*^res^, *Z*^1^, *Z*^2^…, *Z*^6^ are unknown variances.

### Data

**Fig 3:**
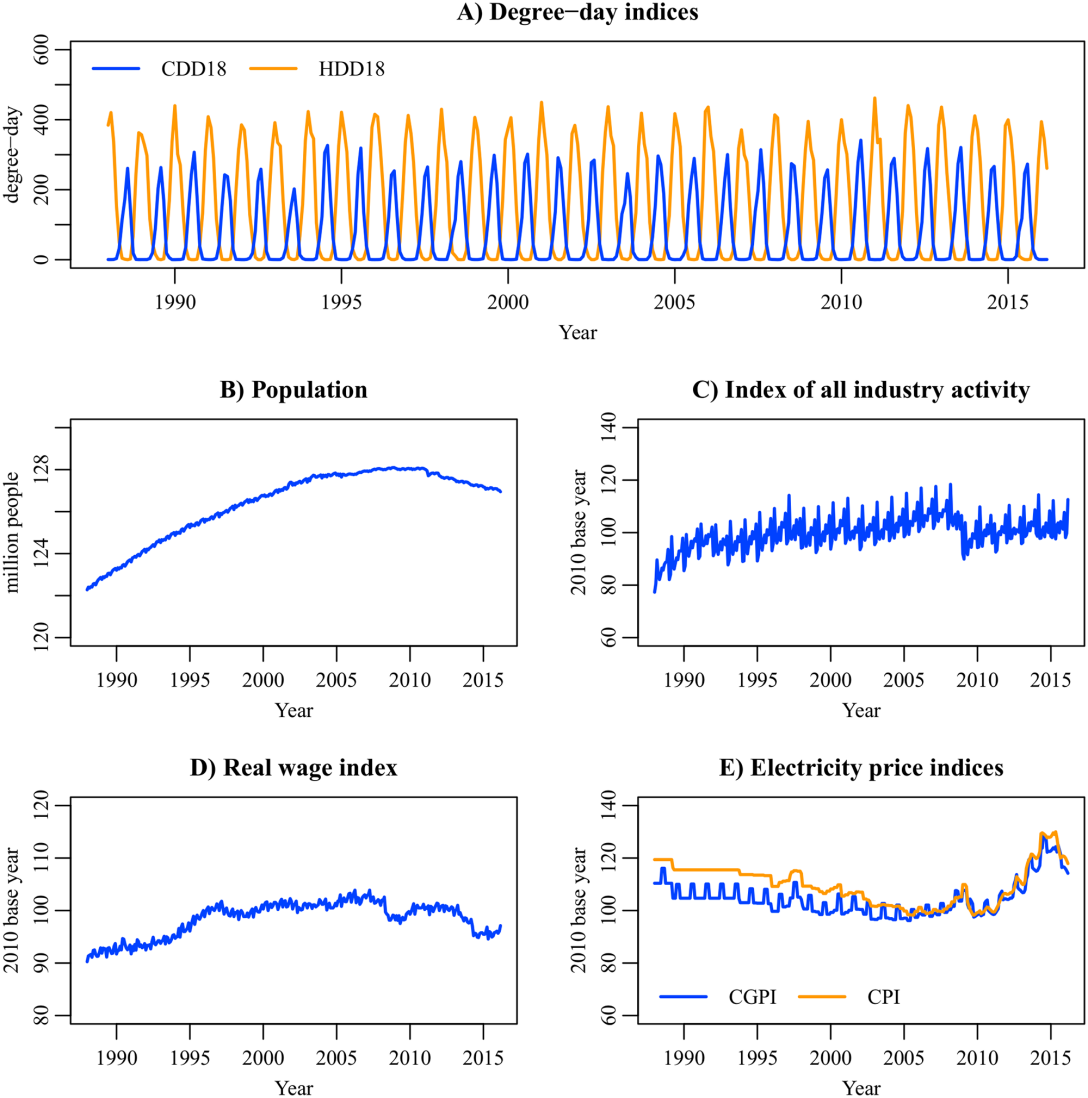
Data of the explanatory variables, January 1988–March 2016. The degree-day indices with the base temperature of 18°C are shown as reference. The DOW and intervention variables are not shown. Data sources are listed in Table 1.

The index of all industry activity (IAA) is used as the economic production index. The IAA is the added-value weighted mean of the production indices in construction, mining, manufacturing, and service industries. The IAA can be interpreted as the monthly GDP. The electricity price indices in the industrial and residential sectors are taken from corporate goods price index (CGPI) and consumer price index (CPI), respectively. The real wage index was calculated by deflating the nominal wage index with CPI (all items except the imputed rent). The base year of the economic indices is 2010. The data sources are listed in Table 1. We use the dataset between January 1988 and March 2016 for model estimation (Fig 3). The data size is 339. The data period was determined based on the data availability. The primary source of the electricity demand data is Energy Survey Statistics [46] provided by Agency for Natural Resources and Energy. As of February 2018, the period of the electricity demand data is January 1986–October 2017, but the IAA data before January 1988 are not available. Moreover, the system of Energy Survey Statistics greatly changed in April 2016 because of the liberalization of electricity retailing. For these reasons, we selected the above data period.

**Table 1:** Model variables and data sources.

Symbol	Variable	Data source
$E_{t}^{\text{ind}},E_{t}^{\text{res}}$	Industrial and residential electricity demands	IEEJ [11]
*N*_*t*_	Population	Statistics Bureau [47]
*C*_*t*_, *H*_*t*_	Cooling and heating degree days	JMA [48], Statistics Bureau [47]
*Q*_*t*_	Index of all industry activity	METI [49]
*Y*_*t*_	Real wage index	IEEJ [11], Statistics Bureau [50]
$P_{t}^{\text{ind}}$	Corporate goods price index (low tension power)	Bank of Japan [51]
$P_{t}^{\text{res}}$	Consumer price index (electricity)	Statistics Bureau [50]
$D_{t}^{1},D_{t}^{2},\ldots ,D_{t}^{7}$	DOW variables	Calculated by the authors
*I*_*t*_	Intervention variable	Calculated by the authors

### Model selection

In the previous section, we defined the model equations of the industrial and residential electricity demands. A number of different models are generated from the model equations depending on (i) the base temperatures of the degree-day indices, (ii) the signs of SEVs (zero or positive), and (iii) the combination of DOW variables. Following previous studies [28, 30, 31], we select the best model based on AIC. AIC of each model is calculated as

$$\begin{array}{c} \text{AIC}=-2L+2k, \end{array}$$ (12)

where *L* is the maximum log likelihood and *k* is the number of unknown parameters. For example, if we assume the fully dynamic model for industrial electricity demand, an observation error variance, seven SEVs, eleven seasonal components, an intercept, and five coefficients need to be estimated from the dataset (see Eq (1)). Hence *k* = 25. Meanwhile, if we assume the SRM for industrial electricity demand, all the SEVs are set to zero. In this case *k* = 18. A smaller value of AIC indicates a better model. To reduce the amount of calculation, we sequentially determine (i)—(iii) rather than simultaneously.

We did not employ the hierarchical likelihood ratio test (HLRT) for model selection. The HLRT attempts to select the best model by iterating the process of applying the likelihood ratio test to two nested models. However, the HLRT has two severe problems [52, 53]. First, the result of model selection can change depending on the order in which candidate models are compared. Second, the HLRT iteratively applies the likelihood ratio test to the same dataset, which inflates the probability of type I error (false positive). When the number of candidate models is large, it is difficult to find the best model by the HLRT.

#### Step (i): Base temperatures of the degree-day indices

First, we determine the base temperatures of the degree-day indices. At this stage, the DOW effect represented by Eq (9) is not included in the observation equation of residential electricity demand. It is assumed that all the SEVs are positive. The base temperature of CDD ($T_{\text{cdd}}^{*}$) is chosen from 18, 19, …, 27°C, and the base temperature of HDD ($T_{\text{hdd}}^{*}$) is chosen from 4, 5, …, 18°C. The lower and upper bounds of the candidate temperatures correspond to 0.1- and 0.9-quantiles of the DAT data in the 47 prefectures (1 January 1988–31 March 2016) [48], respectively. There are 150 (= 10 × 15) candidate models for each of the industrial and residential electricity demands. We calculate AIC for all the candidate models and search the combination $\left(T_{\text{cdd}}^{*},T_{\text{hdd}}^{*}\right)$ that minimizes AIC.

**Fig 4:**
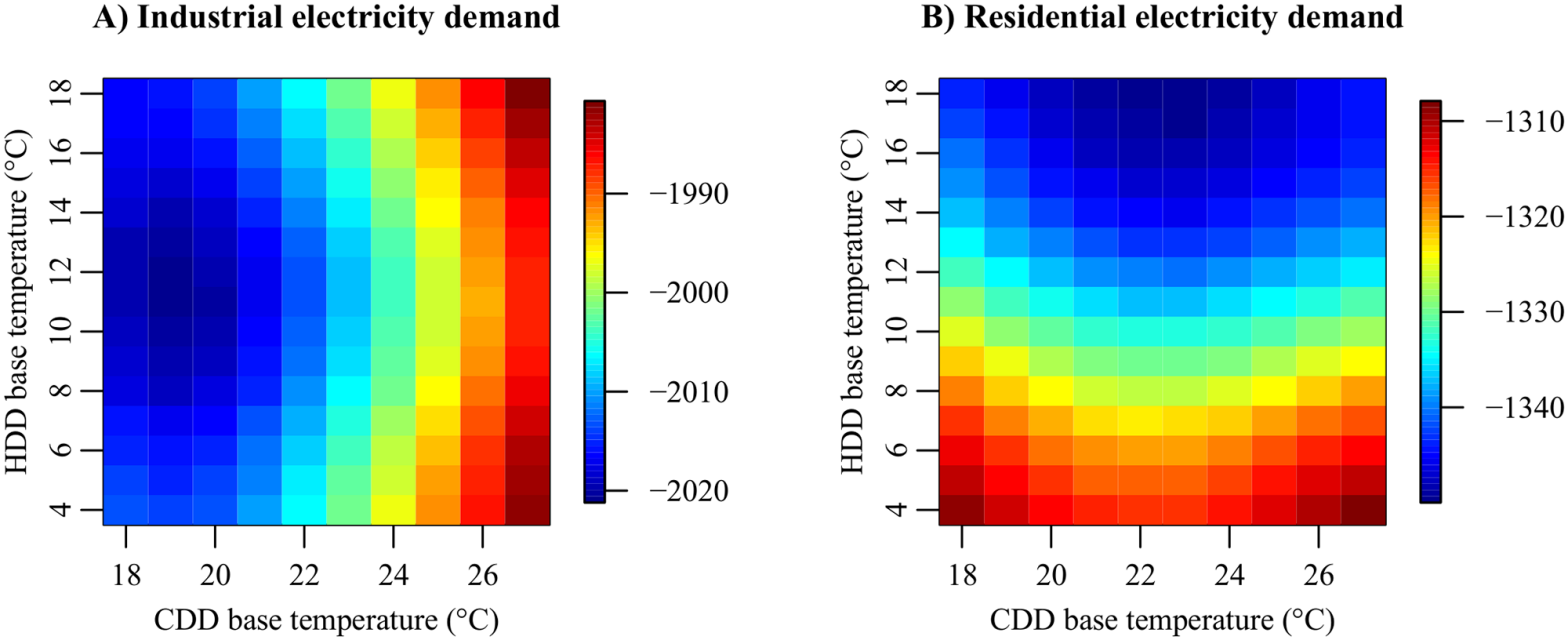
Result of model selection (i): Relationships between the base temperatures of the degree-day indices and AIC of the electricity demand models.

Fig 4 shows the result of model selection. AIC of the industrial electricity demand model is minimized at $\left(T_{\text{cdd}}^{*},T_{\text{hdd}}^{*}\right)=\left(19{}^{\circ}C,11{}^{\circ}C\right)$. AIC of the residential electricity demand model is minimized at $\left(T_{\text{cdd}}^{*},T_{\text{hdd}}^{*}\right)=\left(23{}^{\circ}C,18{}^{\circ}C\right)$. The base temperature of HDD in the industrial sector is much lower than that in the residential sector. This result is explained by the fact that major industries use a large amount of electricity for cooling equipment and goods throughout the year. For example, the information and communication industry must constantly cool down computers in data centers. The retail industry also needs to keep food products cold regardless of the season. As the DAT decreases, the demand for space heating increases, while the demand for cooling equipment and goods decreases. The correlation of electricity demand with the DAT is unclear when the DAT lies between 11°C and 18°C. If the DAT decreases to below 11°C, the correlation becomes visible. Meanwhile, the base temperature of CDD in the residential sector is much higher than that in the industrial sector. This result can be explained by two hypotheses. First, the residential sector may be less sensitive to hot weather than the industrial sector because workers use air conditioners in offices or factories during the daytime on weekdays. Second, consumers’ electricity conservation behavior may lead to high room temperature in summer. In 2005, the Ministry of the Environment launched the Cool Biz campaign to reduce electricity consumption in summer [54–56]. This campaign encourages companies and households to keep the room temperature at 28°C between May 1 and September 30. To prevent heatstroke, workers are recommended to wear light clothes without ties and jackets. According to the online questionnaire survey performed by Mizuho Information and Research Institute in 2015 [57], the Cool Biz was recognized by approximately 80% of respondents (adults living in Japan). Indraganti et al. [55] conducted a field survey on thermal comfort in offices in Tokyo and obtained the result that the comfort temperature for occupants in the summer of 2012 was 27.2°C.

#### Step (ii): Signs of state error variances

Second, we estimate the signs of the SEVs and check whether parameters of the observation equations are time-varying or not. Similar to the step (i), the DOW effect (Eq (9)) is not considered at this stage. The base temperatures of the degree-day indices are set to the optimal levels based on Fig 4. Both of the industrial and residential electricity demand models have seven SEVs. As the ECE is expected to be time-varying from results of previous studies [4, 6–8], we assume *W*^6^ > 0 and *Z*^6^ > 0. There are 64 (= 2^6^) candidate models for each of the industrial and residential electricity demands. We select the best model by minimizing AIC.

Table 2 lists AIC and the signs of SEVs for the top five candidate models. The top five models show similar performance in terms of AIC. This result indicates that electricity demand data can be explained in several different ways. The best model of industrial electricity demand (Ind1) has the dynamic seasonal component, which means that the UEDT follows a stochastic process. The coefficients of the degree-day indices, CGPI, and the intervention variable are time-varying, while the intercept and the coefficient of IAA are constant. The best model of residential electricity demand (Res1) has the static seasonal component. The coefficients of the degree-day indices, CPI, and the intervention variable are time-varying, while the intercept and the coefficient of the real wage index are constant. No stochastic change was detected from the UEDT.

**Table 2:** Result of model selection (ii): Relationships between the signs of state error variances (SEVs) and AIC of the electricity demand models.

**Industrial electricity demand**								
**Model ID**	**AIC**	**Signs of SEVs**						
		*S*^ind^	*W*^1^	*W*^2^	*W*^3^	*W*^4^	*W*^5^	*W*^6^
Ind1	−2024.88	+	0	+	+	0	+	+
Ind2	−2024.56	+	0	+	0	0	+	+
Ind3	−2024.24	+	+	+	+	0	0	+
Ind4	−2023.94	+	+	+	0	0	0	+
Ind5	−2022.88	+	0	+	+	+	+	+
**Residential electricity demand**								
**Model ID**	**AIC**	**Signs of SEVs**						
		*S*^res^	*Z*^1^	*Z*^2^	*Z*^3^	*Z*^4^	*Z*^5^	*Z*^6^
Res1	−1354.14	0	0	+	+	0	+	+
Res2	−1353.65	+	0	+	+	0	+	+
Res3	−1353.31	0	+	+	+	0	0	+
Res4	−1352.80	+	+	+	+	0	0	+
Res5	−1352.71	0	0	+	+	+	0	+

Note: Only top five models are listed. *W*^6^ and *Z*^6^ are positive by definition.

#### Step (iii): DOW variables

Finally, we select the DOW variables which improve AIC of the residential electricity demand model. We applied the stepwise regression to Eq (9). The standard prediction error (SPE) of the Res1 model was used as the response variable (*u*_*t*_). We obtained the result that the numbers of Tuesdays, Thursdays, and Saturdays are statistically significant at the 5% level (Table 3). The number of candidate models which include at least one of the three DOW variables is seven (= 2^3^ − 1). Table 4 lists AIC of the candidate models. The Res1A model, which includes only the number of Tuesdays, has the minimum AIC. AIC of the Res1A model is lower than the Res1 model, which means that the number of Tuesdays contributes to the prediction of residential electricity demand.

**Table 3:** Result of the stepwise regression applied to Eq (9).

DOW variable	Parameter estimate	Standard error	*t* value	*p* value
Tuesday ($D_{t}^{2}$)	−0.526	0.072	−7.332	0.000
Thursday ($D_{t}^{4}$)	0.319	0.082	3.889	0.000
Saturday ($D_{t}^{6}$)	0.234	0.071	3.284	0.001

Notes: The criterion of model selection is AIC. AIC = 894.83, *R*^2^ = 0.149, *F* = 20.8 (*p* = 0.000).

**Table 4:** Result of model selection (iii): Relationships between the DOW variables and AIC of the residential electricity demand model.

Model ID	AIC	Tuesday ($D_{t}^{2}$)	Thursday ($D_{t}^{4}$)	Saturday ($D_{t}^{6}$)
Res1A	−1383.71	1	0	0
Res1B	−1356.67	0	0	1
Res1C	−1356.64	1	0	1
Res1D	−1355.58	1	1	0
Res1E	−1347.28	0	1	1
Res1F	−1332.01	1	1	1
Res1G	−1331.64	0	1	0

Note: The number 1 (0) means that the DOW variable is (not) included in the model.

## Results and discussion

### Results of model estimation

The estimation of the electricity demand models (Ind1 and Res1A) consists of two steps. First, we computed the maximum likelihood estimates of the observation and state error variances (Table 5). Second, we input the estimated variances to the models and computed time variation of parameters with the Kalman filter. This study aims at explaining past electricity demand rather than forecasting future electricity demand. Therefore, we smoothed parameter estimates using the Kalman smoother. As discussed by Petris et al. [29], it is possible to interpret the DLM as a member of Bayesian models. For reference, we numerically estimated the probability distributions of parameters using the MCMC (Markov chain Monte Carlo) method. The estimation results are briefly shown in S1 Appendix.

**Table 5:** Maximum likelihood estimates of observation and state error variances.

Industrial electricity demand		Residential electricity demand	
	Estimate		Estimate
*V*^ind^	8.77076e-05	*V*^res^	9.34049e-04
*S*^ind^	2.95388e-06	*S*^res^	0
*W*^1^	0	*Z*^1^	0
*W*^2^	1.06666e-10	*Z*^2^	1.37188e-09
*W*^3^	2.09909e-11	*Z*^3^	1.68917e-10
*W*^4^	0	*Z*^4^	0
*W*^5^	1.78099e-06	*Z*^5^	3.11607e-06
*W*^6^	1.69895e-05	*Z*^6^	3.22431e-04

Note: *W*^1^, *W*^4^, *S*^res^, *Z*^1^, and *Z*^4^ are zero by definition.

**Fig 5:**
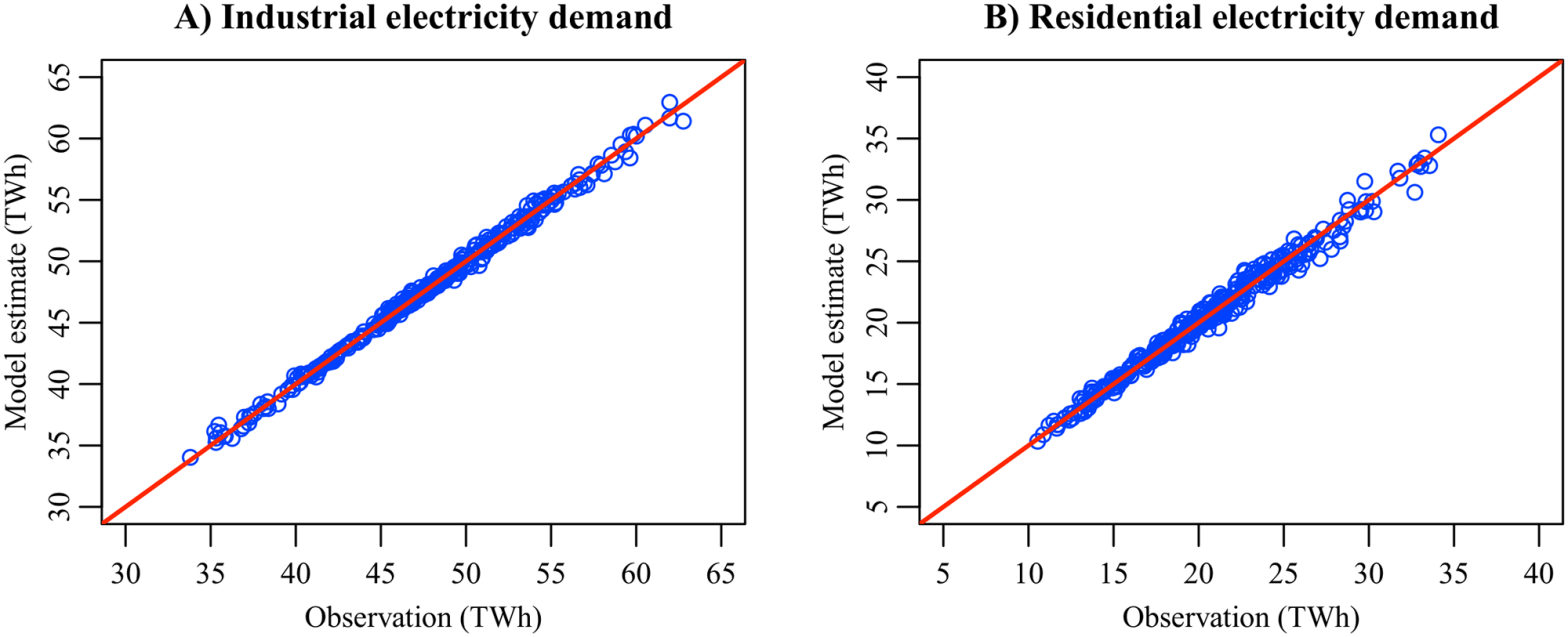
In-sample prediction results of the electricity demand models. The solid line is the 45-degree line.

Fig 5 compares electricity demand data with the model estimates. The plot markers are distributed along the 45-degree line, and no outlier is seen. The MAPEs (mean absolute percentage errors) of the Ind1 and Res1A models are 0.54% and 2.09%, respectively. Our models have the ability to explain historical data of electricity demand.

**Fig 6:**
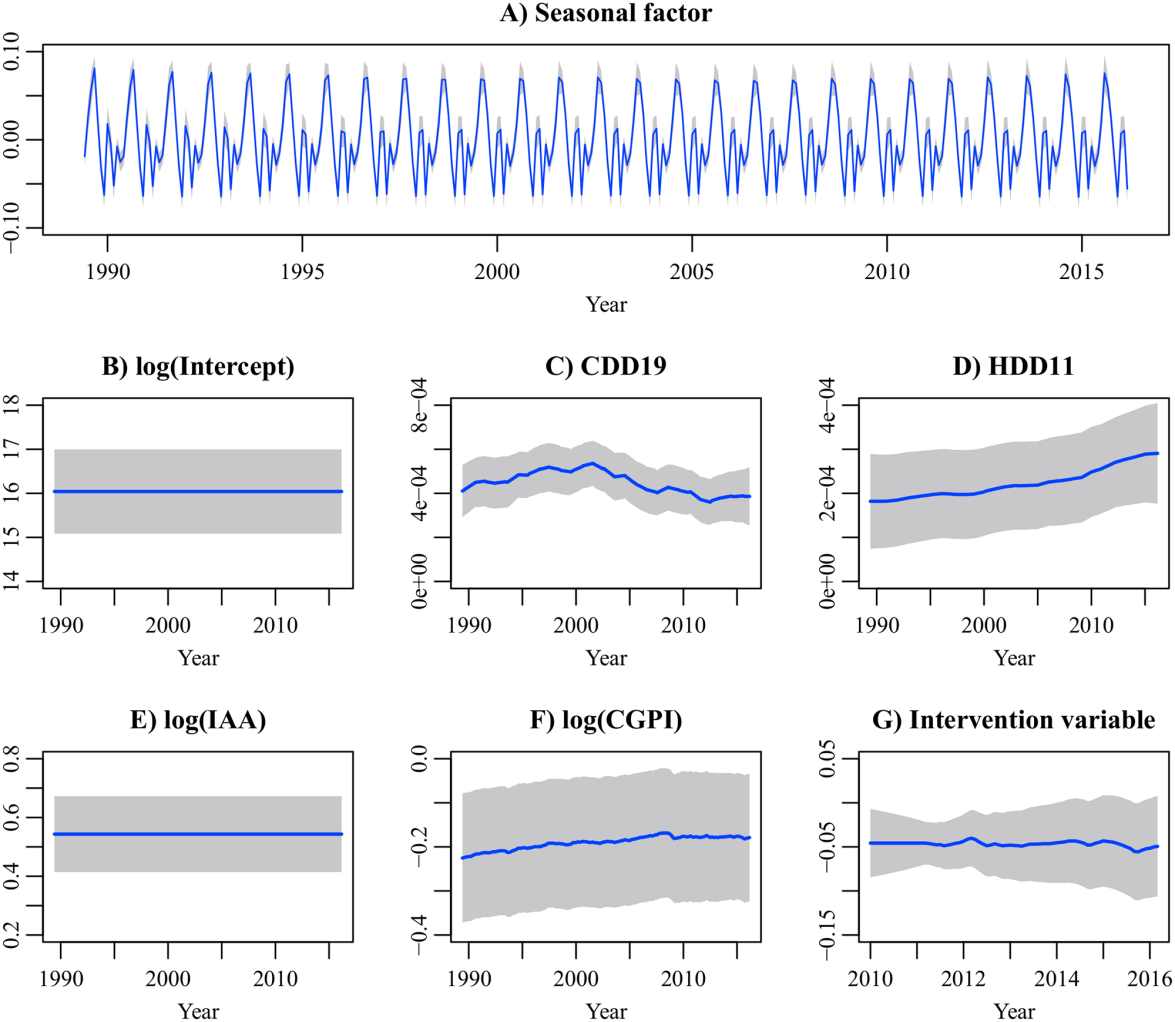
Smoothed parameter estimates of the industrial electricity demand model. The gray regions indicate the 95% confidence intervals. The first 17 estimates corresponding to diffuse initial elements are not shown. For the coefficient of the intervention variable, the estimates between January 2010 and March 2016 are shown.

Fig 6 shows the smoothed parameter estimates of the Ind1 model and their 95% confidence intervals. The Ind1 model has the dynamic seasonal component, which indicates that the underlying electricity demand in each month stochastically changes every year. The coefficient of CDD increased with time but began to decrease in the 2000s. Meanwhile, the coefficient of HDD continued to increase during the period. The coefficients of the degree-day indices determine the amounts of electricity used for cooling and heating under a given DAT. Our result suggests that electricity use for cooling was saved by consumers, while electricity use for heating was not. The coefficient of log(IAA) (production elasticity) is constant. If the IAA increases by 1%, industrial electricity demand increases by 0.54%. The coefficient of log(CGPI) (price elasticity) is time-varying and slowly approaches zero. The price elasticity has a wide confidence interval. An increase in electricity price leads to a decrease in industrial electricity demand, but the effect size is unclear in our dataset. The coefficient of the intervention variable is negative. A structural decrease is observed in industrial electricity demand after the earthquake.

**Fig 7:**
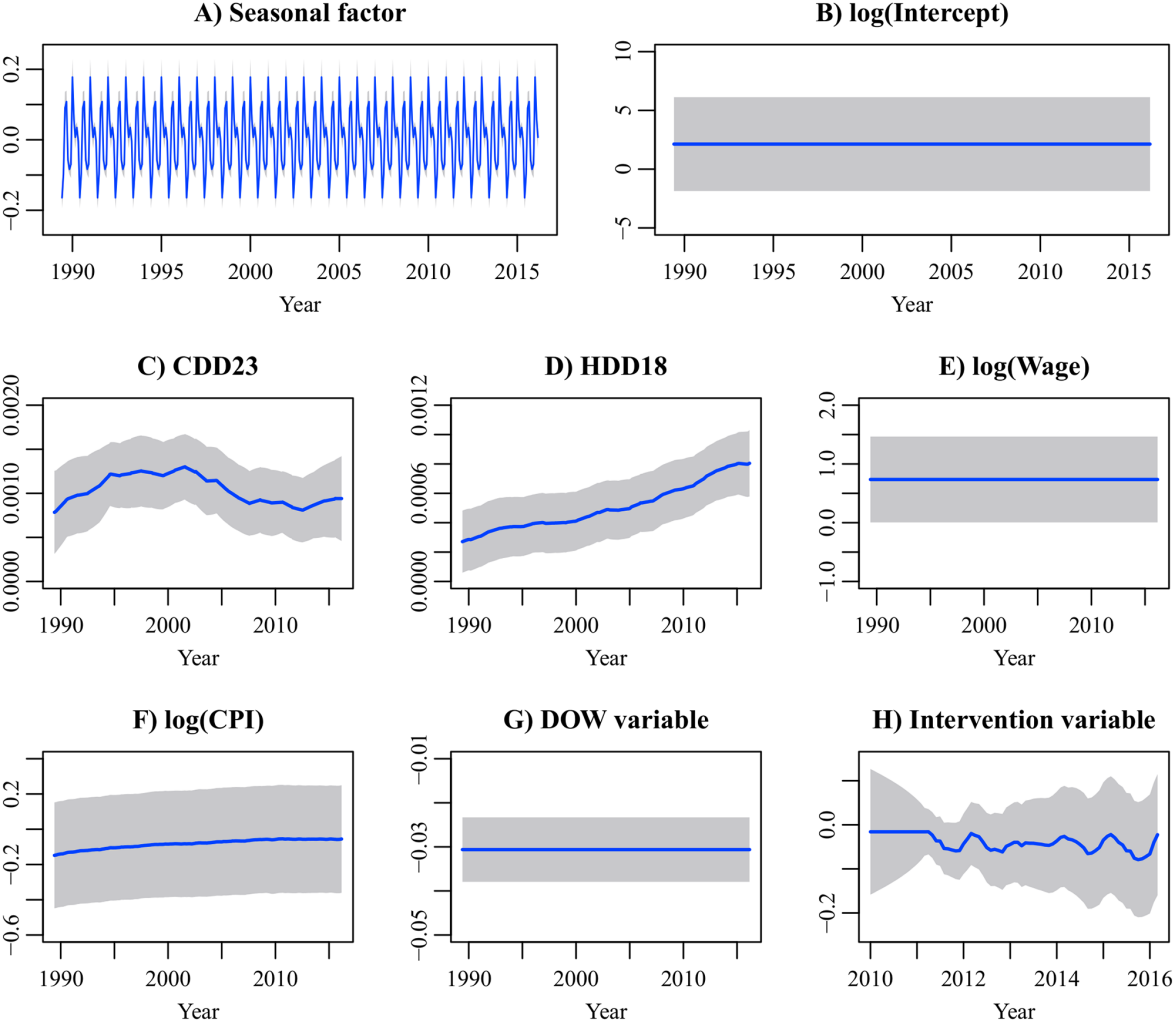
Smoothed parameter estimates of the residential electricity demand model. The gray regions indicate the 95% confidence intervals. The first 18 estimates corresponding to diffuse initial elements are not shown. For the coefficient of the intervention variable, the estimates between January 2010 and March 2016 are shown.

Fig 7 shows the smoothed parameter estimates of the Res1A model and their 95% confidence intervals. The Res1A model has the static seasonal component. The underlying electricity demand in each month was constant throughout the period. The coefficients of the degree-day indices are time-varying and show similar trends to those in the Ind1 model. The CDD coefficient decreased to the level of 1990, while the HDD coefficient continued to increase during the period. The coefficient of log(Wage) (wage elasticity) is constant. If the real wage index increases by 1%, residential electricity demand increases by 0.74%. The coefficient of log(CPI) (price elasticity) is time-varying and slowly approaches zero. The wage and price elasticities have wide confidence intervals, and the effect sizes are unclear in our dataset. The coefficient of the DOW variable is negative. Residential electricity demand on Tuesday tends to be less than that on other days. The coefficient of the intervention variable is negative. A structural decrease is observed in residential electricity demand after the earthquake.

**Fig 8:**
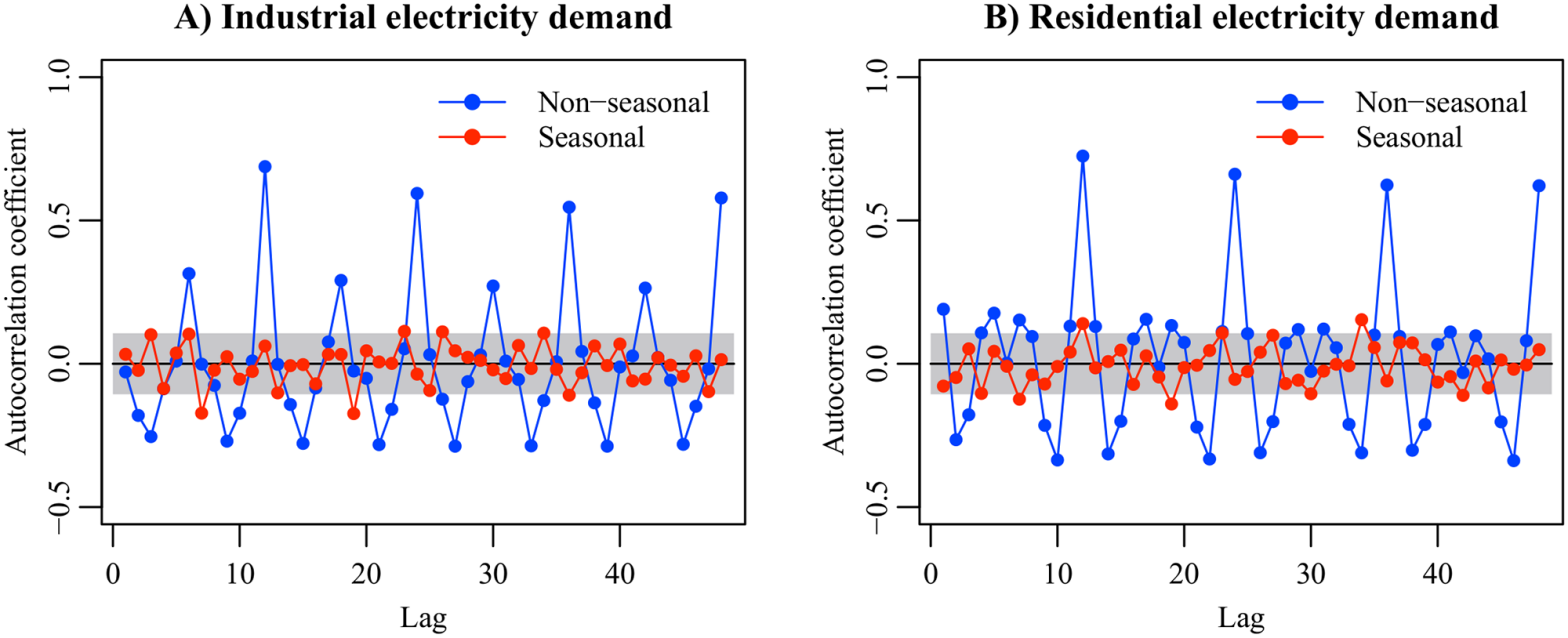
Autocorrelation functions of standardized prediction errors derived from the seasonal and non-seasonal models. The gray regions indicate the 95% confidence intervals.

To understand the role of the seasonal component in predicting electricity demand, we estimated the model which has no seasonal component and compared it with the original model. We focused on the difference between standardized prediction errors (SPEs) of the seasonal and non-seasonal models. The SPE represents time variation of electricity demand which is not explained by the model. Fig 8 shows the autocorrelation functions of the SPEs. The SPEs of the non-seasonal models have positive autocorrelation at lags 12, 24, 36, and 48. This result indicates that electricity demand in each month is close to the level in the same month of past (or future) years. In other words, the industrial and residential electricity demand data contain the seasonal trends with a cycle of 12 months. The seasonal trends are driven by consumers’ habits which are associated with the calendar. For example, public holidays (e.g. Golden Week holidays, Bon holidays, and New Year holidays) change consumers’ activities and create unusual electricity demand patterns. The seasonal component is useful for describing the seasonal trends and contributes to the mitigation of autocorrelation.

### Electricity conservation effect

The definition of the ECE is not self-evident, and previous studies compute the ECE estimates in several different ways. The easiest way is the direct comparison of electricity demands before and after the earthquake. Some previous studies [6–8] set the base year to 2010 in accordance with the governmental guideline for electricity conservation [3]. However, this base-year approach has two disadvantages. First, the estimation result changes depending on the choice of the base year. Second, the estimation result is affected by irrelevant factors because electricity demands in different years are directly compared without controlling the differences in demographic, meteorological, and economic conditions. To avoid these disadvantages, we adopt the intervention-variable approach. The monthly ECE is defined as

$$\begin{array}{c} {\text{ECE}}_{t}=-\frac{E_{t}^{1}-E_{t}^{0}}{E_{t}^{0}}=\frac{\delta_{t}}{E_{t}^{0}}, \end{array}$$ (13)

where $E_{t}^{1}$ is electricity demand with the ECE and $E_{t}^{0}$ is electricity demand without the ECE. $\delta_{t}=E_{t}^{0}-E_{t}^{1}$ is the amount of electricity saved by consumers. The ECE takes a positive value if electricity demand is saved (i.e. $E_{t}^{0}>E_{t}^{1}$). We use the model estimates of electricity demand for the data of $E_{t}^{1}$. The data of $E_{t}^{0}$ are obtained from the model by replacing the intervention variable with the zero vector. By Eq (13), we can compute the ECE estimate which is independent of irrelevant factors. The intervention-variable approach requires no base year, and the estimation result depends only on the structure of the electricity demand model.

**Fig 9:**
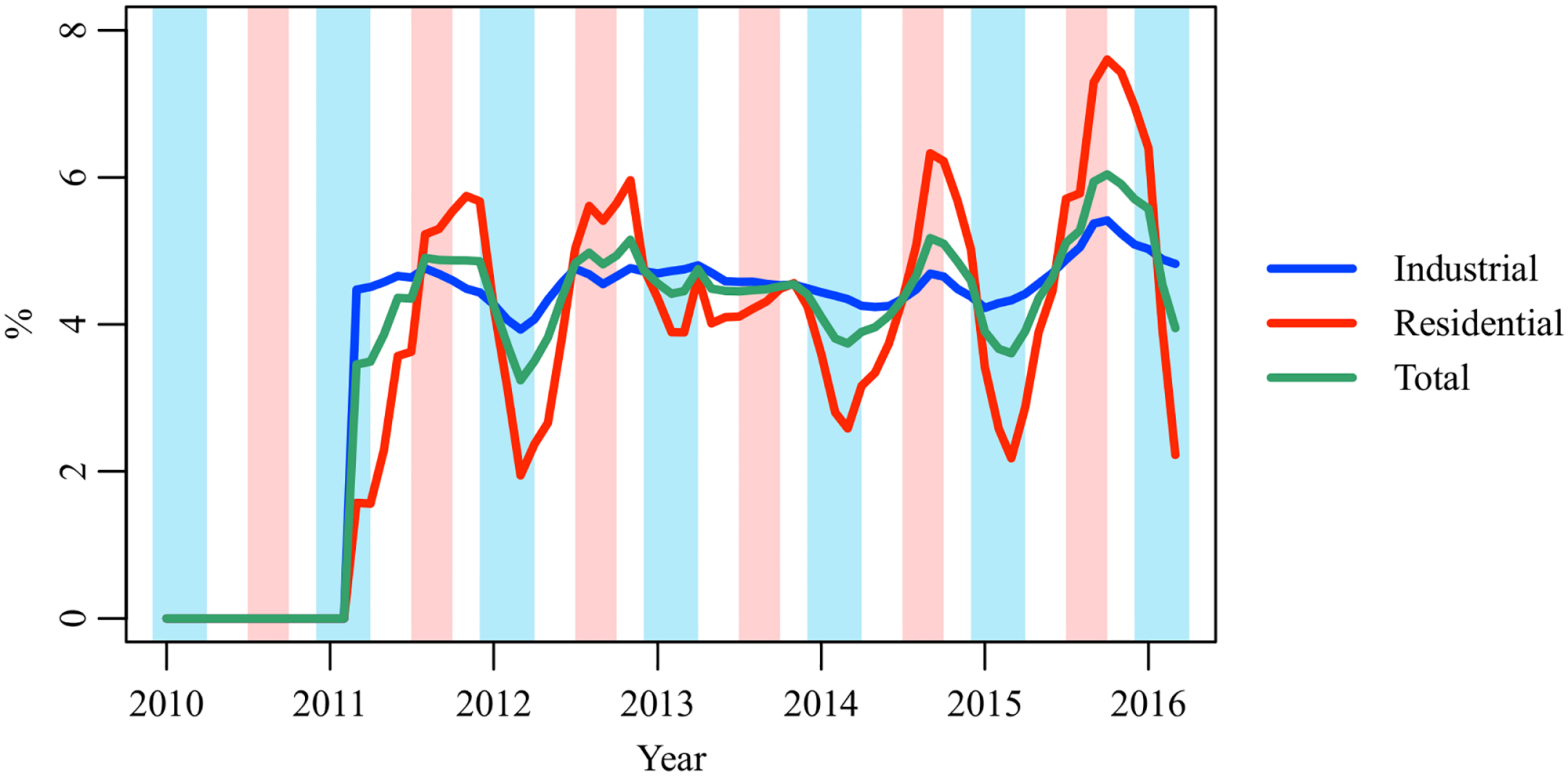
Electricity conservation effects on the industrial, residential, and total electricity demands, January 2010–March 2016. The red region indicates the summer (July–September), and the blue region indicates the winter (December–March).

**Fig 10:**
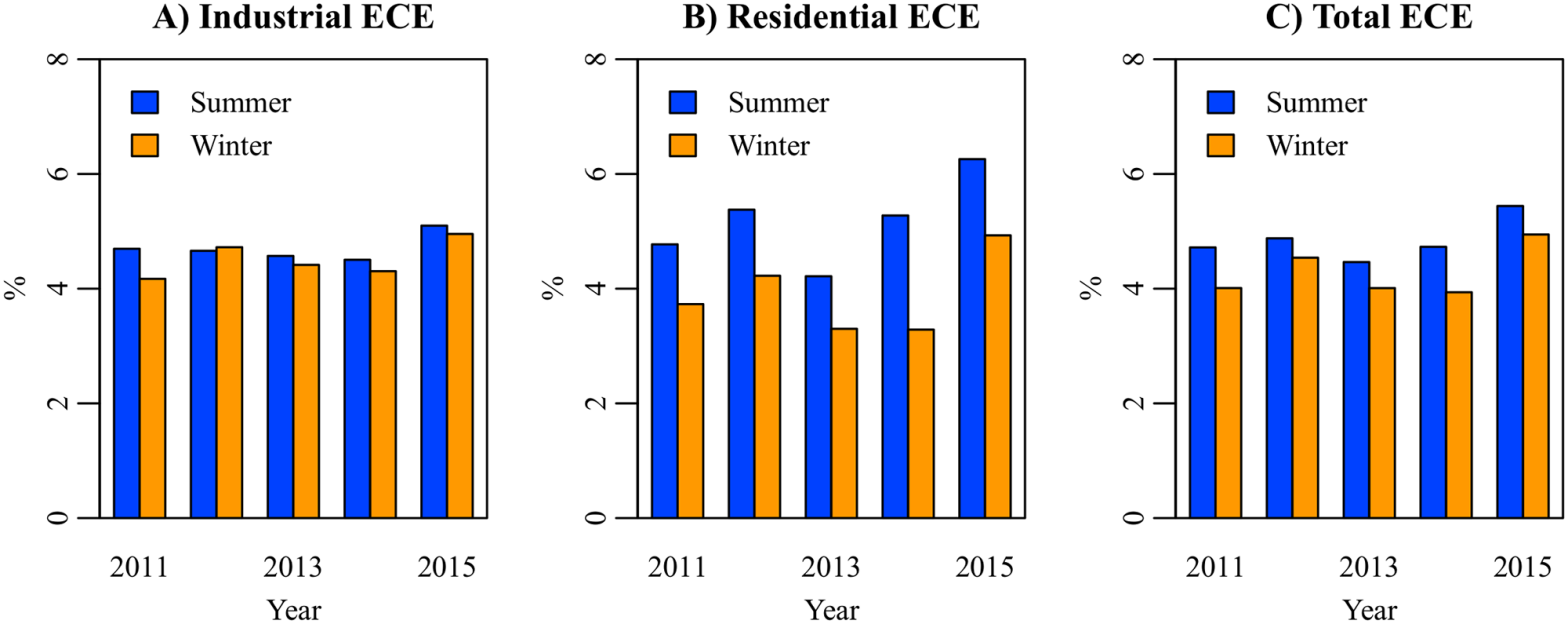
Electricity conservation effects in summers and winters of 2011–2015.

Fig 9 shows the ECEs on the industrial, residential, and total electricity demands between January 2010 and March 2016. The ECEs are expressed in percentage. Since the earthquake in March 2011, the industrial and residential ECEs have been positive. The industrial ECE ranged from 3.9% to 5.4% (mean 4.6%, SD 0.3%). The residential ECE ranged from 1.6% to 7.6% (mean 4.4%, SD 1.5%). The total ECE is estimated at 3.2%–6.0% (mean 4.5%, SD 0.6%). No clear increasing or decreasing trend is observed in the ECE estimates. This result suggests that electricity conservation behavior triggered by the earthquake was not temporary but became established as a habit. Although no legal restriction was imposed on electricity use in households, the residential ECE was close to the industrial ECE. A seasonal trend is observed in the residential ECE. Fig 10 shows the ECEs in summers (July–September) and winters (December–March) of 2011–2015. The residential ECEs in the summers are 0.9%–2.0% higher than the levels in the winters (mean +1.3%, SD 0.4%). A similar trend is also observed in the industrial ECE, but the seasonal difference is relatively small (mean +0.2%, SD 0.2%). S1 Fig shows the correlation between the reduction of electricity demand (*δ*_*t*_) and potential electricity demand ($E_{t}^{0}$). In the industrial sector, *δ*_*t*_ linearly increases as $E_{t}^{0}$ increases. In the residential sector, the correlation is weak, and *δ*_*t*_ randomly distributes in the range of 0.5 TWh–1.5 TWh.

A hypothesis explaining the seasonality of the residential ECE is that Japanese households pay more attention to electricity conservation in summer than that in winter. As shown in Fig 7, the HDD coefficient has an increasing trend, while the CDD coefficient has an inverted U-shaped trend. This result is consistent with our hypothesis. There is another supporting information. To reduce electricity consumption in winter, the Ministry of the Environment launched the Warm Biz campaign in 2005 and encourages companies and households to keep the room temperature at 20°C between November 1 and March 31 [54]. However, the awareness of the Warm Biz is approximately 50%, which is 30% lower than the awareness of the Cool Biz [57]. Further research is needed to prove the seasonality hypothesis.

### Emissions reduction effect of electricity conservation

CO_2_ emissions from power plants are expressed as the product of electricity demand and CO_2_ intensity of electricity (CO_2_ emissions per unit of electricity consumption). Consumers’ electricity conservation behavior after the earthquake decreased electricity demand and contributed to the emissions reduction. Here we estimate the emissions reduction effect of electricity conservation by comparing the emissions in three cases: (1) actual, (2) zero-ECE, and (3) stable electricity supply (SES). First, the emissions in the actual case are expressed in two ways:

$$\begin{array}{c} G_{t}^{1}=S_{t}^{\prime }E_{t}^{\prime }=S_{t}E_{t}. \end{array}$$ (14)

$E_{t}^{\prime }$ is the amount of fossil energy input to power plants, and $S_{t}^{\prime }$ is CO_2_ intensity of the fossil energy. $E_{t}=E_{t}^{\text{ind}}+E_{t}^{\text{res}}$ is the total electricity demand, and *S*_*t*_ is CO_2_ intensity of electricity. $S_{t}^{\prime }E_{t}^{\prime }$ and *S*_*t*_ *E*_*t*_ are the supply-side and demand-side representations of the emissions, respectively. The data of $S_{t}^{\prime }$ and $E_{t}^{\prime }$ were calculated from fuel consumption in electric power companies [11]. Calorific values and CO_2_ intensities of fossil fuels were taken from General Energy Statistics [58]. Second, the emissions in the zero-ECE case are given by

$$\begin{array}{c} G_{t}^{2}=S_{t}\left(E_{t}+\delta_{t}\right)=G_{t}^{1}+S_{t}\delta_{t}. \end{array}$$ (15)

$G_{t}^{2}$ includes the additional emissions (*S*_*t*_ *δ*_*t*_) corresponding to the amount of electricity saved by consumers. Third, the emissions in the SES case are given by

$$\begin{array}{c} G_{t}^{3}=S_{t}^{\text{ses}}\left(E_{t}+\delta_{t}\right). \end{array}$$ (16)

$S_{t}^{\text{ses}}$ is CO_2_ intensity of electricity when nuclear power plants are in operation. For the calculation of $S_{t}^{\text{ses}}$, the equation derived by Honjo and Fujii [59] is useful:

$$\begin{array}{c} S_{t}=\frac{R_{t}S_{t}^{\prime }}{F_{t}}. \end{array}$$ (17)

**Fig 11:**
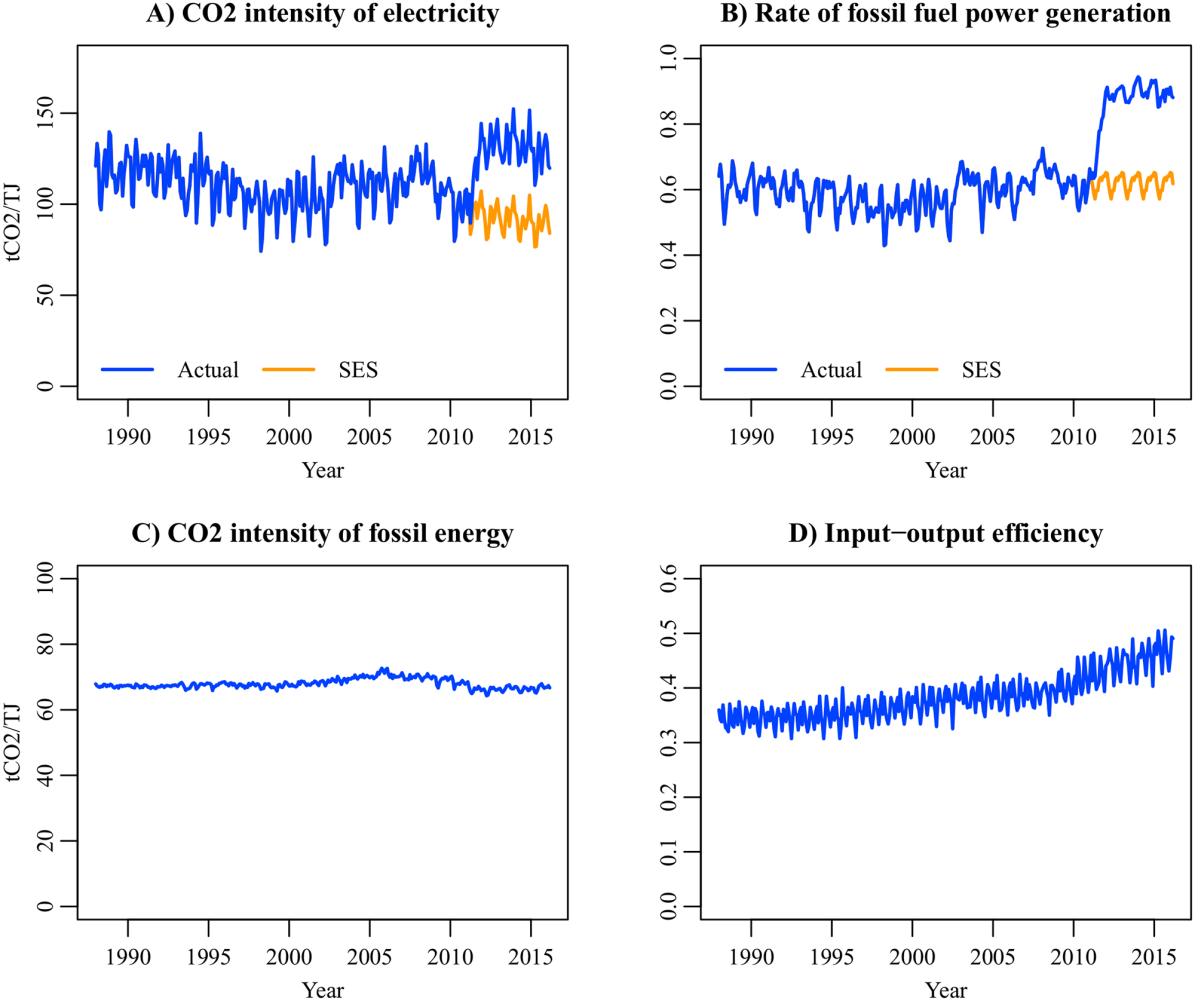
CO_2_ intensity of electricity and its components, January 1988–March 2016. Calculated by the authors from IEEJ [11] and ANRE [58].

*R*_*t*_ is the rate of electricity converted from fossil energy, and *F*_*t*_ is the input-output efficiency of power generation and transmission. The derivation of Eq (17) is summarized in S1 Text. The data of $S_{t}^{\text{ses}}$ were calculated by setting *R*_*t*_ between March 2011 and March 2016 to the average level of 2006–2010. Fig 11 shows CO_2_ intensity of electricity and its components (*R*_*t*_, $S_{t}^{\prime }$, and *F*_*t*_) between January 1988 and March 2016. Due to the shutdown of nuclear power plants, the annual mean of *R*_*t*_ increased from 0.595 in 2010 to 0.910 in 2014. As a result, the annual mean of *S*_*t*_ reached 132.7 tCO_2_/TJ in 2014, which was approximately 1.5 times as high as CO_2_ intensity of coal.

**Fig 12:**
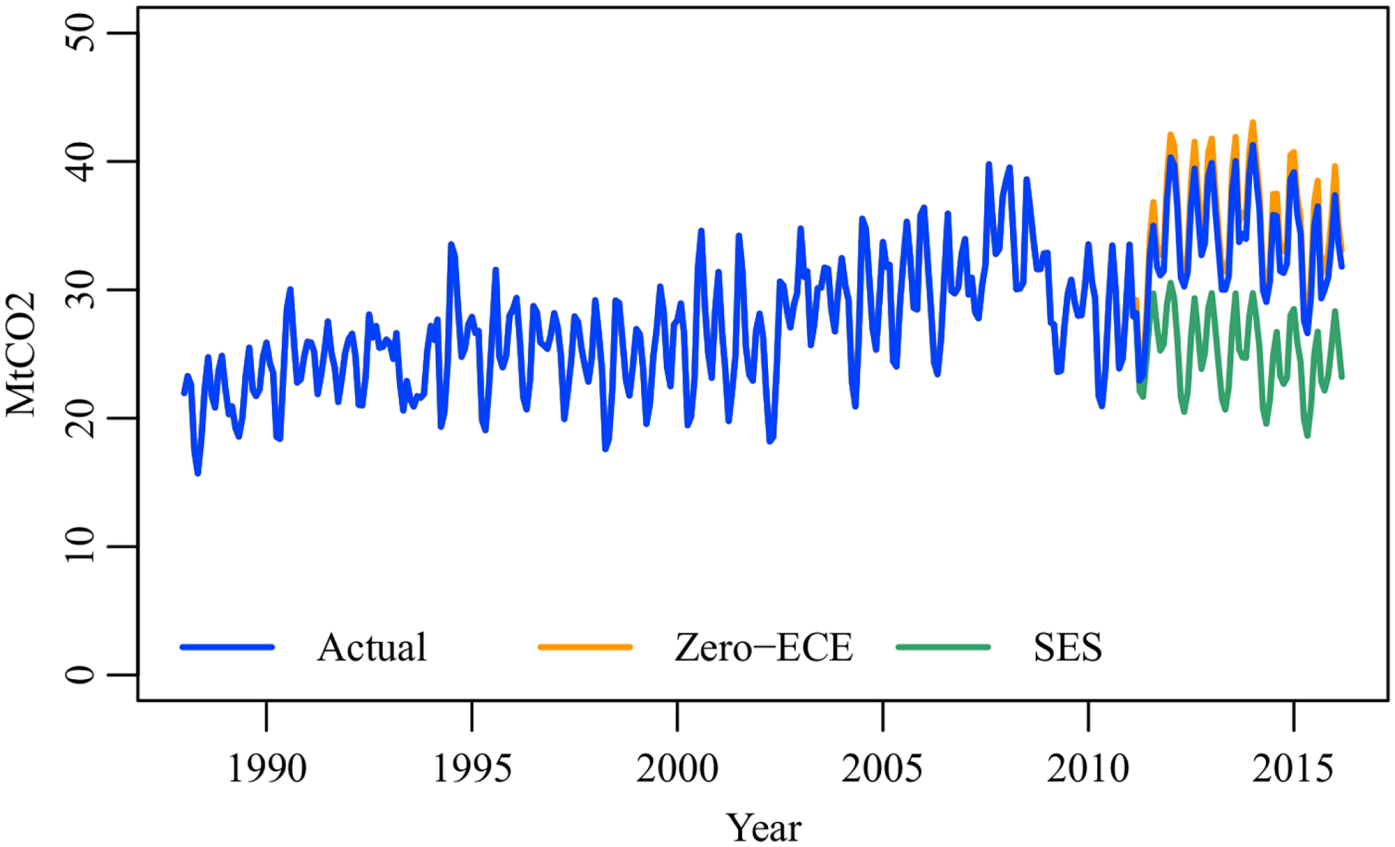
CO_2_ emissions from power plants, January 1988–March 2016.

Fig 12 shows electricity-related CO_2_ emissions in the three cases. The shutdown of nuclear power plants after the earthquake resulted in a rapid increase in the emissions. By $\left(G_{t}^{2}-G_{t}^{3}\right)$, the emissions increase is estimated at 1.49 MtCO_2_–13.31 MtCO_2_ (mean 10.07 MtCO_2_, SD 2.55 MtCO_2_). The emissions in the zero-ECE case were 40.4% larger on average than the emissions in the SES case. This emissions increase was mitigated by consumers’ electricity conservation behavior. By $\left(G_{t}^{2}-G_{t}^{1}\right)$, the emissions reduction effect is estimated at 0.82 MtCO_2_–2.26 MtCO_2_ (mean 1.59 MtCO_2_, SD 0.30 MtCO_2_). The emissions in the actual case were 4.5% smaller on average than the emissions in the zero-ECE case. Our result indicates that the time-varying ECE has a non-negligible impact on the prediction of CO_2_ emissions from electric power companies. Electricity demand models developed before the earthquake are no longer effective and need to be updated.

### Limitations

We conclude this section by discussing three limitations of our study. First, we assumed Gaussian random walk for the ECE to simplify the process of model estimation. This assumption means that time variation of the ECE is purely stochastic and contains no deterministic trend. Our model can evaluate the effect size of electricity conservation but cannot explain the reason why the ECE changes with time. Actually, the stochastic process of the ECE is more complex. Our result suggests that the ECE in the residential sector has seasonality (Fig 10). Previous studies [4, 6–8] report that consumers’ electricity conservation behavior is motivated by various socioeconomic factors: corporate social responsibility, cost reduction, and increased electricity price. Further research about time variation of the ECE is needed. Second, due to a lack of data, we excluded self-consumption of electricity in companies with private power plants. This restriction can cause an over- or under-estimation of the ECE. If a company with constant electricity demand increases self-consumption by 1%, power supply from electric power companies decreases by 1%. In this case, the true ECE is 0%, but our model gives the pseudo-ECE of 1% because self-consumption is unobservable. Self-consumption data are necessary to enhance the accuracy of the ECE estimation. In Japan, unfortunately, it is difficult to access long-term self-consumption data. Third, the ECE in this study represents the aggregate impact of the earthquake on electricity demand. In addition to electricity conservation, the loss of fixed capital stock because of the tsunami also curbed electricity demand in the Tohoku region. However, our model cannot distinguish between the two different effects. This restriction is common in the studies using intervention and dummy variables. For these reasons, the ECE estimates obtained from our model need to be treated carefully.

## Conclusion

In this study, we developed a dynamic linear model of Japan’s monthly electricity demand and estimated time variation of electricity conservation effect (ECE). Our result clearly shows that consumers’ electricity conservation behavior after the Great East Japan earthquake was not temporary but became established as a habit. Between March 2011 and March 2016, the ECE on industrial electricity demand ranged from 3.9% to 5.4% (mean 4.6%, SD 0.3%). The ECE on residential electricity demand ranged from 1.6% to 7.6% (mean 4.4%, SD 1.5%). The reduction of the domestic electricity demand achieved by electricity conservation was estimated at 3.2%–6.0% (mean 4.5%, SD 0.6%). Although no legal restriction was imposed on electricity use in households, the residential ECE was close to the industrial ECE. We found that the residential ECE has seasonality. Between 2011 and 2015, the residential ECEs in summers were 0.9%–2.0% higher than the levels in winters. Using the electricity demand model, we also estimated CO_2_ emissions from power plants. The emissions increase caused by the shutdown of nuclear power plants was estimated at 1.49 MtCO_2_–13.31 MtCO_2_ (mean 10.07 MtCO_2_, SD 2.55 MtCO_2_, +40.4% on average compared to the stable electricity supply case). This emissions increase was mitigated by electricity conservation. The emissions reduction effect was estimated at 0.82 MtCO_2_–2.26 MtCO_2_ (mean 1.59 MtCO_2_, SD 0.30 MtCO_2_, −4.5% on average compared to the zero-ECE case). The time-varying ECE is necessary for predicting electricity demand and CO_2_ emissions in Japan. Japanese policymakers need to update electricity demand models which were developed before the earthquake.

## Supporting information

S1 FigCorrelation between the reduction of electricity demand (*δ*_*t*_) and potential electricity demand ($E_{t}^{0}$).A) *R* = 0.714, *p* = 0.000. B) *R* = 0.295, *p* = 0.021.(PDF)

S1 Text Derivation of Eq (17). (PDF)

S1 Appendix Bayesian estimation of the industrial and residential demand models. (PDF)
