# Supplementary figures and images for: Dynamic linear modeling of monthly electricity demand in Japan: Time variation of electricity conservation effect
Source: PLoS One. 2018 Apr 30;13(4):e0196331. doi: 10.1371/journal.pone.0196331 (PMC5927419; doi:10.1371/journal.pone.0196331)

**A) Industrial electricity demand**

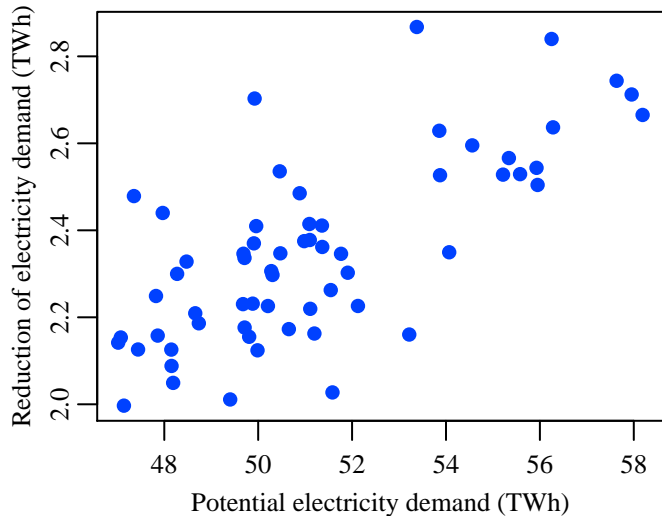

**B) Residential electricity demand**

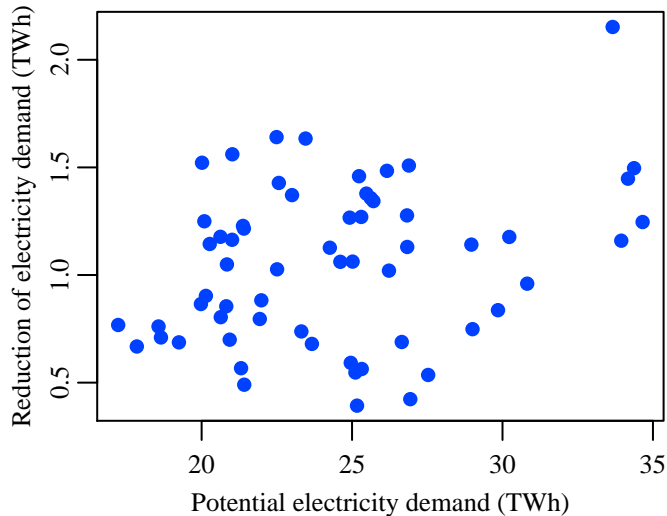

Supplement: S1 Fig — A) R = 0.714, p = 0.000. B) R = 0.295, p = 0.021. (PDF) [file pone.0196331.s001.pdf]
