# Supplementary material for: Dynamic linear modeling of monthly electricity demand in Japan: Time variation of electricity conservation effect
Source: PLoS One. 2018 Apr 30;13(4):e0196331. doi: 10.1371/journal.pone.0196331 (PMC5927419; doi:10.1371/journal.pone.0196331)
Supplement: S1 Text — (PDF) [file pone.0196331.s002.pdf]

S1 Text: Derivation of Eq (17)

Keita Honjo

April 15, 2018

CO_2 intensity of electricity is defined as

$$S = \frac{G}{E}, \tag{1}$$

where G is CO_2 emissions from power plants and E is electricity demand. We classify energy into two types: fossil (type α) and non-fossil (type β). Let E'_i be the amount of energy $i \in \{\alpha, \beta\}$ input to power plants, and let S'_i be CO_2 intensity of energy i. We assume that $S_\beta = 0$. Then

$$S = \frac{S'_\alpha E'_\alpha + S'_\beta E'_\beta}{E} = \frac{S'_\alpha E'_\alpha}{E}. \tag{2}$$

We define the input-output efficiency of power generation and transmission by $F_i = E_i/E'_i$. Following the system of General Energy Statistics developed by Agency for Natural Resources and Energy, we assume $F_\alpha = F_\beta$. The total input-output efficiency is

$$F = \frac{E_\alpha + E_\beta}{E'_\alpha + E'_\beta} = \frac{F_\alpha E'_\alpha + F_\beta E'_\beta}{E'_\alpha + E'_\beta} = F_\alpha. \tag{3}$$

From Eq (2), we obtain the target equation

$$S = \frac{S'_\alpha E_\alpha}{E F_\alpha} = \frac{R_\alpha S'_\alpha}{F}, \tag{4}$$

where $R_\alpha = E_\alpha/E$ is the rate of fossil fuel power generation.
