# Supplementary material for: Dynamic linear modeling of monthly electricity demand in Japan: Time variation of electricity conservation effect
Source: PLoS One. 2018 Apr 30;13(4):e0196331. doi: 10.1371/journal.pone.0196331 (PMC5927419; doi:10.1371/journal.pone.0196331)
Supplement: S1 Appendix — (PDF) [file pone.0196331.s003.pdf]

S1 Appendix: Bayesian estimation of the industrial and residential demand models

Keita Honjo

April 15, 2018

Table 1: Conditions of MCMC experiments

Model equations	Ind1, Res1A
Software	R 3.4.3
Package	*bsts* 0.7.1 [1]
Seed	12345
Number of iterations	10000
Number of burn-in iterations	1000
Gamma prior (shrinkage parameter)	$a = 10^{12}$, $b = 1$

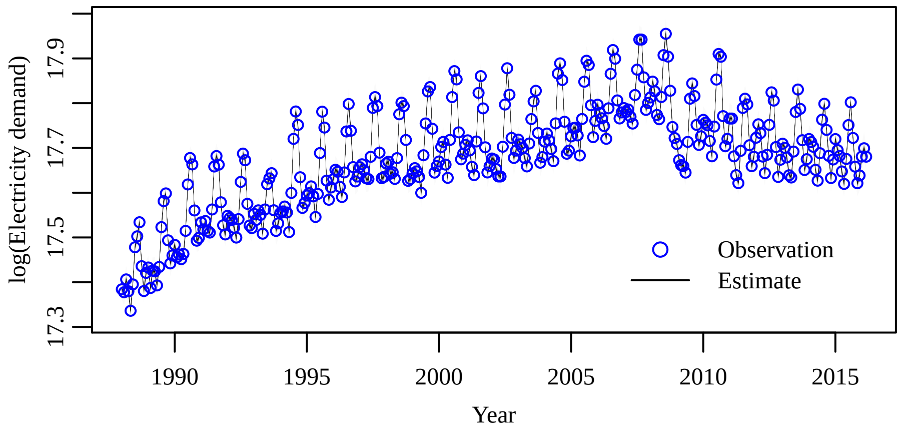

Figure 1: Comparison of the industrial electricity demand data with the MCMC estimates.

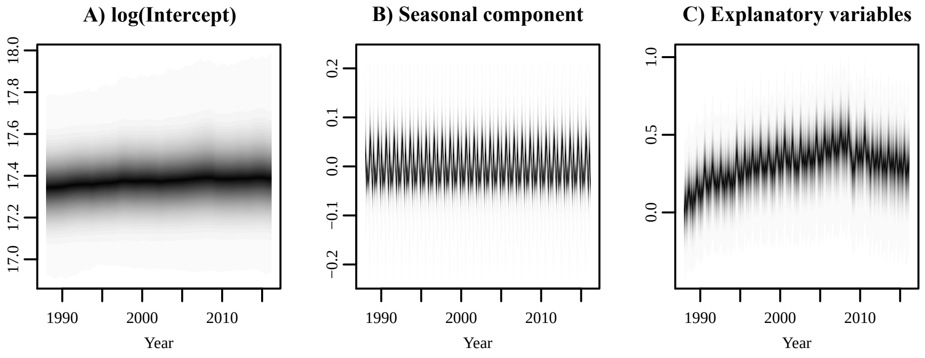

Figure 2: Contributions of the time-varying intercept, seasonal component, and explanatory variables to the MCMC estimates of the industrial electricity demand.

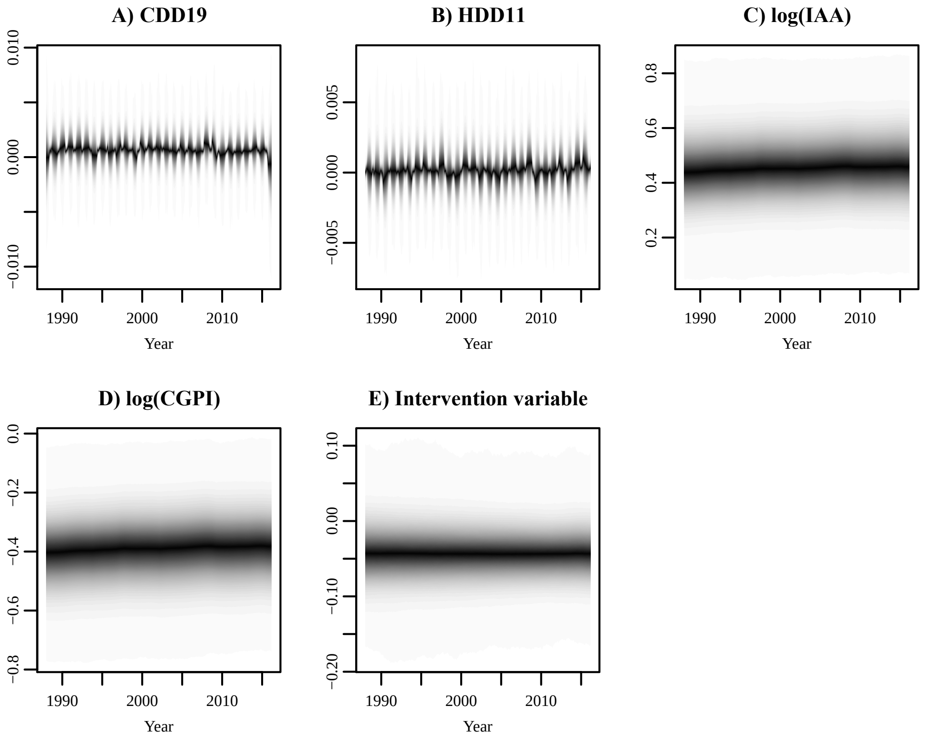

Figure 3: Coefficients of the explanatory variables of the industrial electricity demand model.

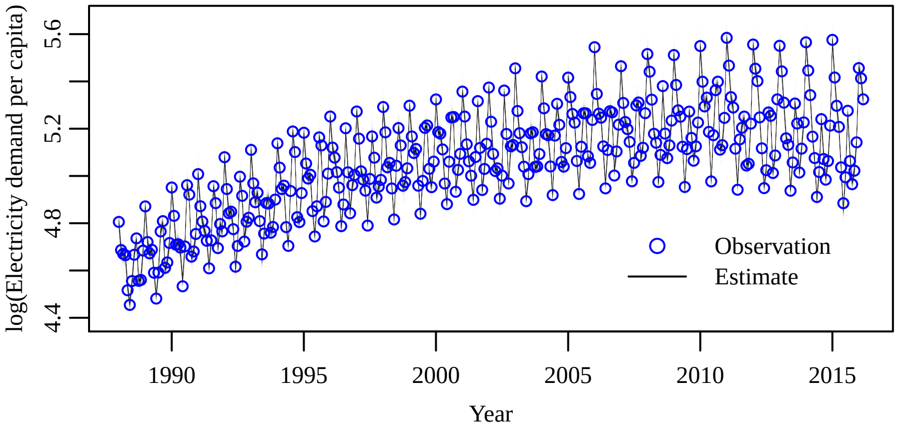

Figure 4: Comparison of the residential electricity demand data with the MCMC estimates.

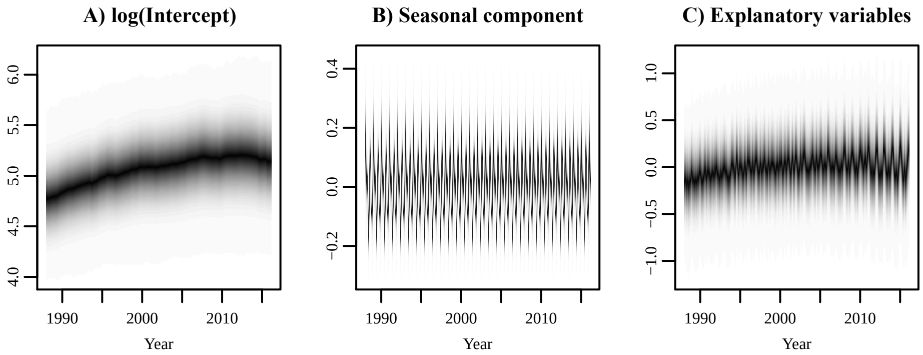

Figure 5: Contributions of the time-varying intercept, seasonal component, and explanatory variables to the MCMC estimates of the residential electricity demand.

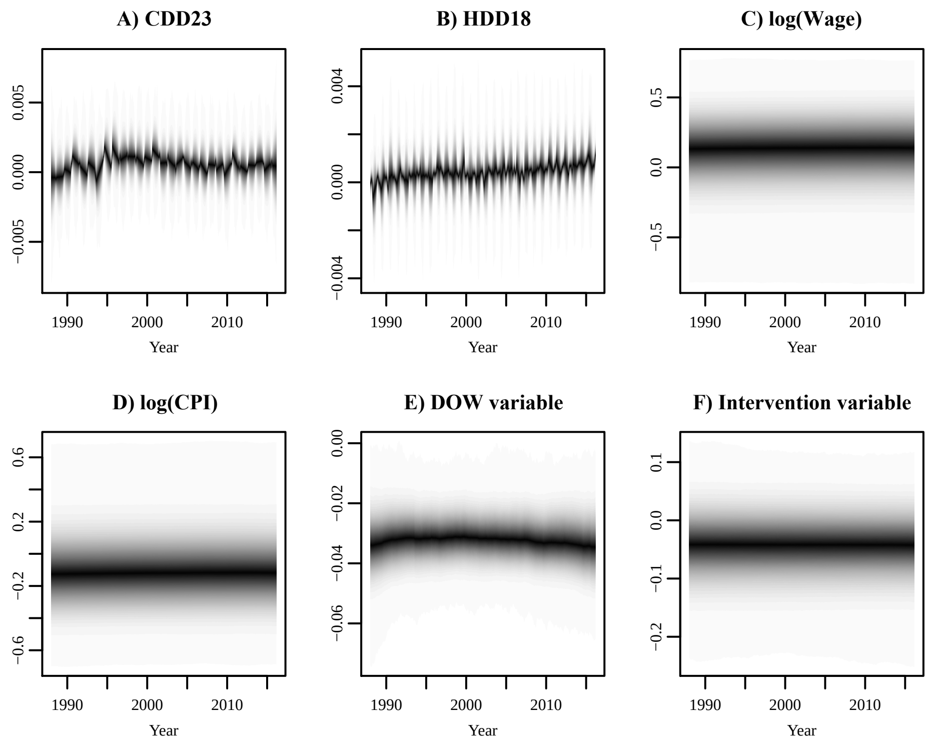

Figure 6: Coefficients of the explanatory variables of the residential electricity demand model.

Reference

1. Scott SL. bsts: Bayesian Structural Time Series; 2017. Available from https://cran.r-project.org/web/packages/bsts/index.html.
